# How Teachers’ Emotional Leadership Influences College Students’ Learning Engagement

**DOI:** 10.3390/bs14090748

**Published:** 2024-08-26

**Authors:** Shuai Wang, Zhuotao Lu, Changjie Li, Yuqiang Zhang

**Affiliations:** 1 Faculty of Education, Qufu Normal University, Qufu 273165, China; 2 Graduate School, University of Jinan, Jinan 250022, China; 3 Institute of Curriculum and Instruction, East China Normal University, Shanghai 200062, China; 52204102003@stu.ecnu.edu.cn (Z.L.); 51184101015@stu.ecnu.edu.cn (C.L.); 4 Research Center for Basic Education Curriculum, Qufu Normal University, Qufu 273165, China; zhangyu7486@qfnu.edu.cn

**Keywords:** teachers’ emotional leadership, learning engagement, achievement emotions, emotional susceptibility, affective events theory

## Abstract

Teachers’ emotional leadership plays a crucial role in influencing college students’ learning engagement. This study, grounded in Affective Events Theory, surveyed 1034 Chinese college students (649 females and 385 males) to examine their perceptions of teachers’ emotional leadership, achievement emotions, emotional susceptibility, and learning engagement. The findings reveal that teachers’ emotional leadership has a significant positive impact on college students’ learning engagement. Specifically, college students’ achievement emotions mediate the relationship between teachers’ emotional leadership and their learning engagement. Additionally, college students’ emotional susceptibility moderates the relationship between teachers’ emotional leadership and their achievement emotions; however, it does not moderate the impact of teachers’ emotional leadership on learning engagement through achievement emotions. This study validates the application of Affective Events Theory in the educational field, deepens the understanding of the mechanism and boundary conditions of emotional leadership’s impact on learning engagement, and offers insights for enhancing college students’ learning engagement.

## 1. Introduction

Learning engagement refers to the quality and quantity of students’ participation and involvement in learning activities [1]. Embedded in action, learning engagement in higher education is often considered from a multidimensional perspective, integrating cognitive, emotional, and behavioral dimensions [2]. Cognitive engagement refers to taking active control over the learning process, utilizing strategies that enhance profound comprehension, and dedicating effort to understand intricate ideas [3]. Emotional engagement focuses on the emotional reactions towards educators, peers, and the academic environment, reflecting the sense of inclusion in and connection to an educational content [3]. Behavioral engagement involves actively participating, exerting effort, maintaining focus, continuing perseverance, displaying positive conduct, and steering clear of disruptive actions. [3]. As an interrelated set of dimensions encompassing students’ cognitive investment, emotional reactions, and active participation in their educational activities, learning engagement is a key factor in observing the learning process and an important predictor of learning outcomes in higher education [4]. Thus, exploring ways to enhance learning engagement is a crucial topic in education research.

College students’ achievement emotions have a significant impact on their learning engagement. Achievement emotions are defined as the positive and negative emotions that students experience and appraise in relation to learning activities or outcomes [5]. For instance, a student may enjoy the challenge of engaging courses that captivate their interest, or feel shame after a poorly executed presentation due to perceived inadequate preparation and anticipated judgment. These experiences are pivotal, as they not only reflect college students’ emotional responses, but also highlight how such emotions can broadly influence their engagement in academic endeavors [5]. Positive achievement emotions, such as enjoyment, hope and pride, are positively correlated with increased engagement in learning activities, whereas, negative achievement emotions, like anxiety and shame, are generally found to negatively impact learning engagement [6].

Learning engagement is correlated with the style of teacher leadership employed. Previous studies have shown that instructional leadership [7] and transformational leadership [8] can directly impact learning engagement. Beyond instructional and transformational leadership, emotional leadership emerges as another pivotal aspect of teacher leadership that can improve the overall performance of schools and students’ learning process [9]. Affective Events Theory (AET) posits that leadership style often affects individuals’ cognition, emotion, and behavior [10]. Based on AET, studies have shown that emotional leadership can improve subordinates’ work engagement [11] and job performance [12]. Therefore, teachers’ emotional leadership may be an important antecedent of learning engagement.

Emotional leadership refers to leaders who can understand and manage their own emotions and the emotions of others to inspire and direct them towards achieving expected goals [13]. In educational settings, teachers play a crucial role as leaders in emotional expression and regulation [14]. For instance, during a challenging lecture, a teacher alleviates students’ anxiety by remaining patient and showing care, thereby reducing their stress and guiding their learning. Teachers’ emotional leadership emphasizes the capacity to recognize and manage both their own and their students’ emotions to effectively motivate and guide educational outcomes. This type of teacher leadership often involves elements of emotional intelligence such as empathy, self-awareness, and social skills [15]. Some studies have pointed out that teachers’ emotional intelligence is related to specific aspects of learning engagement. For example, the empathy displayed by teachers can inspire students in post-secondary education to ask questions proactively and engage with the course material [16], and teachers’ social skills used in the classroom can affect the emotional and behavioral engagement of students in grades five to ten [17]. Although studies have highlighted the connection between teachers’ emotional intelligence and various aspects of learning engagement, the broader role of teachers’ emotional leadership in shaping the emotional dynamics within classroom settings and its overall impact on college students’ learning engagement remains relatively unexplored.

AET provides a theoretical foundation for exploring emotional dynamics. According to AET, (a) specific events, such as leadership styles and behaviors, serve as emotional triggers that elicit individuals’ affective reactions; (b) these reactions, resulting from individuals’ cognitive appraisal of specific events, drive subsequent working outcomes (e.g., behaviors and attitudes); (c) these working outcomes are influenced by both the specific events and the affective reactions, and (d) individual traits not only moderate affective reactions to specific events but also influence the relationship between specific events and working outcomes through these affective reactions [18].

In the context of higher education, AET can help us understand how teachers’ emotional leadership influences students’ emotions and learning engagement. The teaching and learning process is full of emotions, and teachers’ emotional actions and interactions affect the emotional states and responses of students [19]. Based on AET, when teachers display emotional leadership, they create specific affective events that elicit college students’ affective reactions. These affective reactions can be understood as achievement emotions, as they result from cognitive appraisals that involve evaluating the significance of the specific events caused by teachers’ emotional leadership [5]. Learning engagement, including interrelated cognitive, emotional, and behavioral dimensions, represents the learning outcomes of college students. It is influenced not only by achievement emotions, but also by teachers’ emotional leadership. Therefore, achievement emotions may mediate the relationship between teachers’ emotional leadership and learning engagement. Furthermore, the effect of teachers’ emotional leadership has certain boundary conditions due to the different individual traits among college students. For example, when a teacher delivers an engaging lecture by showing enthusiasm and creating an inspirational learning environment, some students may resonate with the emotions and be deeply affected by the teacher, while others may remain unresponsive and stay calm. This interpersonal phenomenon can be attributed to differences in emotional susceptibility, which is an individual trait referring to individuals’ sensitivity to emotional information and the intensity of their emotional reactions [20]. Thus, emotional susceptibility may moderate college students’ achievement emotions in response to teachers’ emotional leadership, but also influence the relationship between teachers’ emotional leadership and learning engagement through their achievement emotions.

In summary, the purpose of the study is to explore the association between teachers’ emotional leadership, college students’ achievement emotions, emotional susceptibility, and learning engagement, and to provide evidence for how teachers’ emotional leadership influences college students’ learning engagement. The research questions of this study are:

1. What impact does teachers’ emotional leadership have on college students’ learning engagement?
2. Do achievement emotions mediate the impact of teachers’ emotional leadership on college students’ learning engagement?
3. Does emotional susceptibility moderate the impact of teachers’ emotional leadership on college students’ achievement emotions?
4. Does emotional susceptibility moderate the impact of teachers’ emotional leadership on college students’ learning engagement through achievement emotions?

Using an online survey platform, this study gathered 1034 responses from Chinese college students. The independent variable was teachers’ emotional leadership, the dependent variable was learning engagement, achievement emotions served as the mediator, and emotional susceptibility was the moderator. Regression analysis methods were employed to examine the impacts of teachers’ emotional leadership on learning engagement, the mediating role of achievement emotions, and the moderating role of emotional susceptibility. The contributions of this study are as follows. First, to our knowledge, existing studies have rarely focused on the mechanisms between teachers’ emotional leadership, achievement emotions, emotional susceptibility, and learning engagement; this study is one of the initial efforts to examine these mechanisms. Second, by applying AET in higher education and conducting empirical tests, this study not only helps to validate the universality and applicability of AET across different fields, but also underscores the importance of emotional dynamics in educational practice. Additionally, this study can provide a reference for higher education institutions, helping educators better understand and manage students’ emotional experiences, thereby improving learning outcomes and the quality of education.

## 2. Literature Review and Research Hypotheses

### 2.1. Teachers’ Emotional Leadership and College Students’ Learning Engagement

Emotional leadership involves the process whereby leaders strategically manage and influence the emotions of organization members through emotional intelligence, emotional contagion, and constructive communication, creating a positive atmosphere and effectively advancing the achievement of common organizational goals [21,22,23,24]. To break down this process, we can identify several key dimensions that constitute emotional leadership. These dimensions encompass the abilities required to recognize and manage emotions, influence others emotionally, and effectively interact within the organization. Specifically, the dimension of emotional leadership includes emotional cognition and management abilities (e.g., emotional intelligence), emotional influence (e.g., emotional support), and interactive capabilities (e.g., communication abilities, conflict resolution abilities). Based on the concept of emotional leadership, this study defines teachers’ emotional leadership as the process in the teaching context whereby teachers, through emotional intelligence, emotional contagion, and constructive communication, strategically manage and mobilize college students’ emotions to create a positive learning atmosphere, thereby achieving the desired learning outcomes jointly anticipated by teachers and college students. Teachers with emotional leadership skills identify, understand, and respond to college students’ emotional needs during learning activities, and utilize their own emotions to influence college students’ learning. Accordingly, the aspects of teachers’ emotional leadership equally encompass emotional cognition and management abilities, emotional influence, and interactive capabilities.

Learning engagement is a contentious concept [25,26]. Bond et al., in their systematic review on learning engagement, defined it as the energy and effort students invest in their learning community, observable through a range of cognitive, emotional, or behavioral indicators [27]. Learning engagement first occurs at the individual student level. Accurately analyzing and measuring learning engagement at this level cannot be sufficiently performed by merely observing behavioral manifestations; it also necessitates considering their cognitive and emotional aspects [28]. Therefore, in this study, we consider learning engagement to encompass cognitive, emotional, and behavioral dimensions.

Teachers’ emotional leadership plays a crucial role in the learning environment. Existing studies have focused on specific aspects of teachers’ emotional leadership to explore its relationship with learning engagement. In terms of emotional recognition and management abilities, it has been shown that teachers with high emotional intelligence can better perceive, understand, and manage students’ emotions, which significantly fosters college students’ emotional and cognitive development, thereby encouraging greater engagement, increased motivation, and enhanced capacity for effective self-management of learning [29]. As for emotional influence, teachers who provide emotional support by showing concern, kindness, and care for students’ attitudes, emotions, and behaviors can increase their behavioral engagement [30]. Regarding interactive capabilities, teachers with strong social and emotional skills can more effectively manage classrooms, foster positive environments, and guide students in resolving conflicts [31]. This, in turn, helps enhance college students’ motivation, participation, and academic performance.

Although existing studies have recognized the association between specific aspects of teachers’ emotional leadership and students’ learning engagement, they have not comprehensively explored the impact of emotional leadership as an integrated concept on learning engagement. This indicates that the specific relational dynamics between teachers’ emotional leadership and students’ learning engagement require further detailed investigation. According to AET [18], teachers’ emotional leadership acts as an affective event in learning activities, and learning engagement represents the learning outcomes of college students. By enhancing students’ emotional satisfaction and positivity, teachers’ emotional leadership can boost college students’ attention to and interest in course content (cognitive engagement), foster positive feelings and emotional participation in learning (emotional engagement), and motivate college students to participate more frequently in learning-related activities (behavioral engagement). Thus, we propose that teachers’ emotional leadership may promote the learning engagement of college students.

### 2.2. The Mediating Role of Achievement Emotions

Achievement emotions are the range of emotional responses experienced by students when facing learning activities and achievement outcomes, directly associated with students’ motivation, engagement, and academic performance [32]. According to the control–value theory, achievement emotions can be classified into four main types based on the dimensions of emotional valence (positive or negative), activation (activating or deactivating), and object focus (related to the activity or outcome), forming positive activating, positive deactivating, negative activating, and negative deactivating emotions [33]. Achievement emotions significantly impact college students’ learning experiences and performance. Positive activating emotions, such as enjoyment, hope, and pride, reflect a positive appraisal by college students of learning activities and their outcomes, and can stimulate their interest in learning, enhance intrinsic motivation, and promote active participation in the learning process [5]. Positive deactivating emotions, such as relief and relaxation, represent a positive appraisal of learning and may reduce college students’ motivation or immediate drive in specific situations, but help maintain long-term energy and commitment [5]. Negative activating emotions, such as anger, anxiety, and shame, reflect a negative appraisal and, although they may reduce intrinsic motivation and interest, can also produce strong extrinsic motivation, relying on external regulation to focus more on the task [5]. Negative deactivating emotions, such as boredom and hopelessness, exhibit a negative appraisal and often lead to distraction and a lack of willingness to invest effort [5].

A body of research has indicated a direct connection between specific aspects of teachers’ emotional leadership and college students’ achievement emotions. Firstly, Goldman and Goodboy found that teachers’ emotional support and validation can enhance college students’ emotional interest and elevate their positive emotional valence, highlighting the crucial role of teacher emotional support in influencing college students’ emotional outcomes in the classroom [34]. Moreover, teachers’ emotional leadership fosters harmonious teacher–student relationships [35], which enhance college students’ sense of support and control, thereby reducing negative activating emotions such as anxiety and shame, as well as negative deactivating emotions like hopelessness [36]. Further, teachers, through effective communication, can identify and meet students’ emotional needs, and alleviate the emotional regulation and management needed for college students to adapt to the classroom environment and meet teachers’ expectations, directly promoting positive achievement emotions like enjoyment, hope, and pride during the learning process [37], while reducing negative achievement emotions such as anger, anxiety, shame, hopelessness, and boredom [38].

Current research reveals significant links between college students’ learning engagement and achievement emotions as well. Achievement emotions are considered to be a key factor in driving college students’ cognitive, emotional, and behavioral engagement. For example, Sadoughi and Hejazi indicate that college students’ positive achievement emotions play a key role in stimulating their learning engagement [39]. More specifically, Linnenbrink notes that positive achievement emotions are positively correlated with cognitive engagement. When college students experience positive emotions, they are more likely to engage in deep thinking, maintain focused attention, and participate actively in learning activities [40]. Robinson and colleagues observed that college students exhibiting positive activating emotions displayed heightened levels of behavioral engagement. Similarly, high levels of behavioral engagement were also associated with college students who reported high positive deactivating emotions [41].

To summarize, prior research has already addressed the relationship between specific aspects of teachers’ emotional leadership and achievement emotions, as well as the link between achievement emotions and learning engagement. However, there is a paucity of discussion on whether teachers’ emotional leadership influences learning engagement through achievement emotions. Based on AET [18], it can be considered that the influence of teachers’ emotional leadership on college students’ learning engagement is mediated through achievement emotions. Specifically, the emotional leadership of teachers, such as providing emotional support and recognizing and managing college students’ emotions, constitutes a key affective event in the learning environment. Such events directly impact college students’ emotional responses, including stimulating positive achievement emotions (such as enjoyment, hope, pride) and reducing negative achievement emotions (such as anxiety, shame, hopelessness). Achievement emotions, as direct emotional responses to affective events, further affect college students’ attitudes and behaviors. In this way, we expect that teachers’ emotional leadership may enhance college students’ learning engagement through their achievement emotions.

### 2.3. The Moderating Role of Emotional Susceptibility

Emotional susceptibility refers to the sensitivity of individuals to perceive, respond to, and adapt to external emotional stimuli. This trait determines how and to what extent a person is influenced when faced with others’ emotional expressions [42]. There are significant differences in emotional susceptibility among individuals, which may be caused by various factors including genetic background, gender, and early experiences [20]. Studies indicate that individuals with high emotional susceptibility are highly attuned to their own emotional changes, exhibiting strong reactions, and are adept at interpreting others’ emotions, often mimicking their expressions, voices, and postures; moreover, these individuals place significant value on interpersonal relationships, emphasize interdependence, and are particularly attentive to others, resulting in their emotional experiences being substantially influenced by external feedback [43,44]. Focusing on the college student population, Frisby demonstrated that college students with high emotional susceptibility establish closer relationships with teachers, more easily perceive the emotional support provided by teachers, and are more easily influenced by positive emotions around them, leading them to hold a more positive evaluation of the classroom environment [45].

According to AET, emotional responses as well as the understanding and experience of affective events are influenced by individual traits [18]. College classrooms serve as a potential arena for emotional expression and communication, where the emotional interaction between teachers and college students naturally develops and deepens, influencing the learning process [45]. In such emotionally active learning environments, the emotional states and behavior patterns of teachers significantly affect college students’ emotions, achieving the direct transmission of emotions from teachers to college students and proving the phenomenon of emotional contagion between them [46]. Emotional contagion refers to the tendency of individuals to automatically imitate and synchronize another person’s facial expressions, voice, postures, and movements, thereby achieving emotional uniformity [47] (p. 7). During the process of emotional contagion, emotions may transfer from the individual expressing the emotions to another individual mimicking these emotions. This means that when teachers display specific emotions (such as happiness, sadness, anger, etc.), the observing or interacting college students may transfer these emotions to themselves, beginning to exhibit similar emotional states [45,48]. However, it is important to note that emotional contagion does not occur to the same degree among all individuals, due to different levels of emotional susceptibility among individuals [43].

According to the relationship between teachers’ emotional leadership and achievement emotions (see Section 2.2), existing research has primarily focused on the direct impact of emotional leadership on achievement emotions, lacking a discussion on how emotional leadership influences achievement emotions. Combining discussions on the phenomenon of emotional susceptibility and emotional contagion, we propose that emotional susceptibility, as an individual trait, shows diversity among different college students. This individual difference at the level of emotional susceptibility has a moderating effect on the effectiveness of teachers’ emotional leadership. Specifically, for college students with high emotional susceptibility, they are more likely to notice and absorb the emotional information transmitted by teachers’ emotional leadership, thereby experiencing stronger emotional responses and higher changes in achievement emotions. Conversely, college students with low emotional susceptibility may be less sensitive to the perception of teachers’ emotional leadership, resulting in weaker emotional responses and lower changes in achievement emotions.

### 2.4. The Research Hypotheses

Based on AET, teachers’ emotional leadership may impact college students’ learning engagement. However, the impact of teachers’ emotional leadership on learning engagement may not be solely direct, but could also be mediated by college students’ achievement emotions. Furthermore, college students’ emotional susceptibility may moderate the effect of teachers’ emotional leadership on their achievement emotions. Consequently, the mediating effect of emotional leadership on learning engagement could be contingent upon the level of emotional susceptibility.

**Figure 1:**
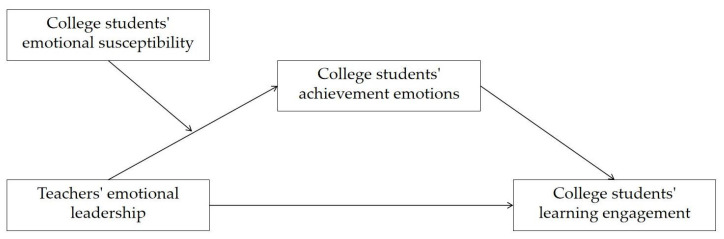
Research model.

In summary, the following hypotheses (see Figure 1) are proposed:

**H1.**  *Teachers’ emotional leadership has a significant positive effect on college students’ learning engagement;*

**H2.**  *Achievement emotions mediate the relationship between teachers’ emotional leadership and college students’ learning engagement;*

**H3.**  *Emotional susceptibility plays a moderating role in the effect of teachers’ emotional leadership on college students’ achievement emotions;*

**H4.**  *The mediating effect of teachers’ emotional leadership on college students’ learning engagement is moderated by emotional susceptibility.*

## 3. Materials and Methods

### 3.1. Data Collection and Participants

The study was approved by the Ethics Committee of University of Jinan (approval number UJN-YGB-2024-001). Informed consent was obtained from all participants prior to their inclusion in the study. Detailed explanations regarding the purpose, procedures, potential risks, and benefits of the study were provided via email. Participants were assured of their right to withdraw from the study at any time without penalty. To ensure confidentiality, all personal identifiers were removed from the data. Data collection in this study was conducted through an online platform called WJX. This platform is a professional survey collection platform in China, servicing clients that have covered 90% of higher education institutions in China. Given the demographic diversity of participants on WJX, it is a reliable channel for reaching as many Chinese college students as possible. Stratified random sampling was employed to select the participants. Initially, Chinese provincial administrative regions were divided into East, Central, and West based on levels of economic development. Subsequently, one representative provincial administrative region was randomly selected from each area. Within the chosen provinces, three municipal administrative units were selected randomly, taking into account regional differences such as population size and economic indicators to ensure a representative sample. Specifically, a list of all municipalities within each selected province was compiled, and three municipalities were chosen using a random number generator. Within each selected municipality, universities were categorized by their types (e.g., research-oriented, teaching-oriented) and size (e.g., large, medium, small). A proportional random sampling method was then applied to select universities, ensuring representation across different types and sizes. This was done by first listing all universities in each municipality, then categorizing them based on the aforementioned criteria, and finally using random number generation to select a proportionate number of universities from each category. Finally, students from both natural sciences and humanities and social sciences disciplines were randomly chosen as participants, proportionate to the number of universities in each municipality.

To ensure the eligibility of the survey participants, the following conditions were set: participants must be Chinese college students currently enrolled and the institutions must be located within China. Only users who met the above criteria were able to fill out the questionnaire. The required sample size was estimated using G*Power, selecting the multiple linear regression model of Fixed model, with R^2^ deviation from zero. The parameters included an effect size f^2^ of 0.02, α = 0.05, and a power (1 − β) of 0.95, with three predictors. This analysis indicated a necessary total sample size of 863. However, it is important to acknowledge potential biases due to non-response, as certain groups of respondents might be underrepresented. For instance, if less motivated students or those with lower academic performance are less likely to respond, this could lead to an overestimation of overall learning engagement. To mitigate these biases, follow-up reminders were sent to encourage higher response rates. Considering the response rate of online surveys, approximately 1200 questionnaires were initially distributed via the WJX platform to the selected universities, based on an estimated distribution efficiency of about 70%. The researchers completed the ethical review in late January 2024. The survey was open for completion from late January to February 2024. To ensure the integrity of the responses, participants were encouraged to complete the survey independently. Additionally, incentives such as small gift cards were offered to encourage participation. The survey was designed to be anonymous to protect the confidentiality of the participants and to encourage honest and unbiased responses. In the end, 1161 participants submitted their responses through the WJX platform. Among the submitted samples, two types were considered invalid. One type was those with a very short completion time. In the submitted samples, over 95% of participants spent more than 100 s browsing and answering the question. Some participants, however, submitted their questionnaires in just 52 s, which was not enough time to complete the entire surrey. Therefore, samples with a completion time of less than 100 s were considered invalid [49]. Another type was where participants provided the same answer for every item, such as rating every item with a value of 1. This behavior suggests a possible reliance on satisficing to expedite the completion of the survey rather than a genuine engagement with and contemplation of each question’s content [50]. A total of 127 invalid samples were identified and discarded, accounting for approximately 10.94% of the total responses. The initial sample size calculation indicated a need for at least 863 participants to achieve the desired statistical power. After discarding responses that did not meet the validity criteria, the final valid sample count stood at 1034, surpassing the initially required minimum. This adjustment ensures a robust analysis and enhances the reliability of our findings.

In the sample, there were 649 females, accounting for 62.77%, and 385 males, accounting for 37.23%. In terms of age distribution, the majority of the participants were between 17 and 19 years old, accounting for 739 students. This was followed by 219 students aged between 20 and 22 years, 62 students aged between 23 and 25 years, and a smaller group of 14 students aged 26 years and above. There were 507 students from natural science majors, representing 49%, and 527 from humanities and social science majors, representing 51%. Additionally, participants were drawn from universities under three categories: 21 students from universities identified as “Double First-Class” (an initiative targeting top Chinese universities and disciplines), 634 from provincial key universities (typically more established, resource-rich, and academically reputable), and 379 from other provincial universities (generally newer with fewer resources and lower academic standing).

### 3.2. Instruments

All the scales/questionnaires used in this study adopted a 5-point Likert-type, with 1 representing “strongly disagree” and 5 representing “strongly agree”.

Teachers’ emotional leadership was measured using Jin’s revised Emotional Competence Scale [51], which has been used to measure the core emotional trait of leadership and has shown good reliability and validity. This scale has been widely used in previous research. For example, Wan et al. employed the same scale to measure the impact of emotional leadership on subordinates’ work engagement, reporting a Cronbach’s alpha of 0.98 and passing the discriminant validity test [11]. The authors translated the English items into Chinese, and the translations were reviewed by two bilingual translators, followed by a formal back-translation process. They compared the back-translated version with the original English items to ensure semantic and conceptual equivalence. Discrepancies were discussed and resolved through consensus, ensuring the accuracy and appropriateness of the final Chinese version. This rigorous validation process enhances the reliability of the translation. Additionally, the revision involved an update to the item wording to better reflect contemporary theoretical understandings of teachers’ emotional leadership, and the inclusion of items that target specific emotional competencies relevant to educational settings. Each modification was based on a consultation with field experts to ensure the scale’s relevance and accuracy in measuring the intended constructs. For example, the original item “Accurately reads peoples’ moods, feelings or nonverbal cues” [51] was revised to “The teacher can accurately grasp the students’ emotional state, psychological feelings, and non-verbal information such as body language”. The scale contains seven questions. The Cronbach’s alpha in the study was 0.973, and the corrected item-total correlations (CITCs) of the items in the scale were above 0.8. Furthermore, there were significant correlations among the items (see Appendix A). When administering the survey, participants were instructed to consider their general experiences with teachers and courses rather than focusing on a specific instructor or course. This approach was also applied to other scales/questionnaires used in the study, ensuring that the responses reflected college students’ overall perceptions and experiences in their educational environment rather than reactions to a particular course or professor.

Achievement Emotions were measured using the short version of the Achievement Emotions Questionnaire (AEQ-S), revised by Bieleke et al. [52]. The AEQ-S is a well-established instrument known for its high reliability and validity in measuring achievement emotions in educational research [52]. The AEQ-S covers three situational contexts: class-related emotions, learning-related emotions, and test emotions. For this study, learning-related emotions were selected as the measurement items based on the study’s context and objectives. This questionnaire has been widely used in previous research, consistently demonstrating good reliability and validity. For instance, Gao and Yang employed the same questionnaire to investigate the mediating role of negative emotion intensity between trait emotional intelligence and emotion regulation strategies, reporting a Cronbach’s alpha of 0.89 [53]. The questionnaire was localized and reviewed. For example, the original item “I am so happy about the progress I made that I am motivated to continue studying” [52] was revised to “You are very happy about the progress you have made, which motivates you to continue learning” due to the tendency of Chinese individuals to be more reserved and less likely to use the first person to describe their own state. The questionnaire consists of 32 questions. The Cronbach’s alpha in the study was 0.963, and the CITCs of the items in the questionnaire were above 0.6. Likewise, there were significant correlations among the items (see Appendix B).

Emotional Susceptibility was measured using the Chinese version of the Emotional Contagion Scale (ECS) revised by Wang et al. [54]. This revision was necessitated by cultural differences in emotional expression and perception. The Chinese version of the ECS was adapted by modifying some original items that are more applicable to the Chinese cultural context. For example, the original item “If someone I’m talking with begins to cry, I get teary-eyed” [20] was revised to “If someone I’m talking with begins to cry sadly, I feel sad and my eyes get teary” to reflect the cultural emphasis on interpersonal harmony and the specific context of sadness in Chinese culture [54]. These changes were validated through a pilot study involving Chinese participants, which confirmed the scale’s reliability and validity in this new setting. This scale has been widely used in previous research and has shown good reliability and validity. For example, Wan et al. employed the same scale to investigate the moderating role of emotional susceptibility between emotional leadership and positive emotions, reporting a Cronbach’s alpha of 0.803, and this also passed the discriminant validity test [12]. The scale contains 13 questions. The Cronbach’s alpha in the study was 0.970, and the CITCs of the items in the scale were above 0.7. There were significant correlations among the items (see Appendix C).

Learning Engagement was measured using a questionnaire adapted from Reeve’s study, which has shown strong psychometric validity and high internal consistency [55]. This questionnaire has been extensively employed in educational research, including by Jang et al., who confirmed its strong reliability and validity in their study of Korean high school students’ learning engagement [56]. Reeve’s questionnaire encompasses four dimensions: cognitive engagement, emotional engagement, behavioral engagement, and agentic engagement. In the current study, learning engagement is conceptualized to include cognitive, emotional, and behavioral dimensions. Therefore, items measuring cognitive engagement, emotional engagement, and behavioral engagement from Reeve’s questionnaire were selected. Likewise, the questionnaire was localized and reviewed. For example, the original item “When I’m in this class, I listen very carefully” [55] was revised to “You listen very carefully in class” to better align with the participants’ cultural and linguistic habits. The questionnaire in the study consists of 14 questions. The Cronbach’s alpha in the study was 0.977, and the CITCs of the items in the scale were above 0.8. Additionally, there were significant correlations among the items (see Appendix D).

### 3.3. Data Analysis

SPSS 22 was utilized for statistical analysis. The reliability of the scales/questionnaires was evaluated using reliability analysis. Additionally, correlation and regression analyses were conducted to test the hypotheses and to investigate the interrelations among the independent variable of teachers’ emotional leadership, the mediating variable of achievement emotions, the moderating variable of emotional susceptibility, and the dependent variable of learning engagement.

## 4. Results

### 4.1. Normality Test

Four variables, including teachers’ emotional leadership, achievement emotions, emotional susceptibility, and learning engagement, were created. Each of these variables was derived from the simple mean of corresponding item scores. Kline posits that if the absolute values of kurtosis are less than 10 and the absolute values of skewness are less than 3, the data can be considered approximately normally distributed [57] (p. 77). According to the results of the normality test (see Table 1), the absolute values of kurtosis and skewness for the four variables were within acceptable ranges, indicating that the data in this study were approximately normally distributed.

### 4.2. Correlation Analysis

Pearson correlation coefficients were computed to assess the relationships among variables. As shown in Table 2, learning engagement was positively related to teachers’ emotional leadership (*r* = 0.395, *p* < 0.01), achievement emotions (*r* = 0.277, *p* < 0.01), and emotional susceptibility (*r* = 0.371, *p* < 0.01). Additionally, teachers’ emotional leadership was positively related to achievement emotions (*r* = 0.229, *p* < 0.01) and emotional susceptibility (*r* = 0.383, *p* < 0.01). Furthermore, achievement emotions were positively related to emotional susceptibility (*r* = 0.213, *p* < 0.01).

### 4.3. Direct Effect Test

From Table 3, with teachers’ emotional leadership as the independent variable and learning engagement as the dependent variable for linear regression analysis, the direct effect size of teachers’ emotional leadership on learning engagement was 0.264 (*p* < 0.01), indicating that teachers’ emotional leadership has a significant positive impact on learning engagement. Hypothesis 1 was thus supported.

### 4.4. Mediating Effect Test

**Figure 2:**
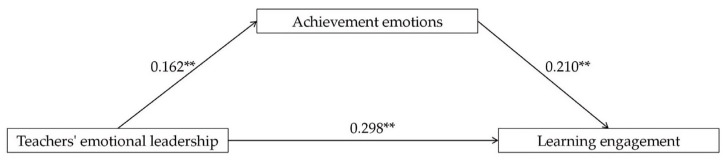
Mediation model. ** *p* < 0.01.

According to Table 3, the effect of teachers’ emotional leadership on achievement emotions was significant (effect size = 0.162, *p* < 0.01), indicating that teachers’ emotional leadership can significantly enhance college students’ achievement emotions. The effect of achievement emotions on learning engagement was also significant (effect size = 0.210, *p* < 0.01), suggesting that achievement emotions can significantly promote college students’ learning engagement. To test the mediating effect, we calculated the indirect effect of teachers’ emotional leadership on learning engagement through achievement emotions (a × b). The indirect effect was 0.034, with a standard error of 0.010 and a confidence interval of [0.027, 0.066], which was statistically significant (*p* < 0.01). This indicates that achievement emotions partially mediate the relationship between teachers’ emotional leadership and learning engagement. Additionally, the direct effect of teachers’ emotional leadership on learning engagement was 0.264 (*p* < 0.01), and the total effect was 0.298 (*p* < 0.01). Given that the direct impact of teachers’ emotional leadership on learning engagement remained significant but was reduced when achievement emotions were considered, this further confirmed the presence of a partial mediation effect. Hypothesis 2 was supported. To visually present the mediating effect, a mediation model diagram was constructed (see Figure 2).

### 4.5. Moderating Effect Test

**Figure 3:**
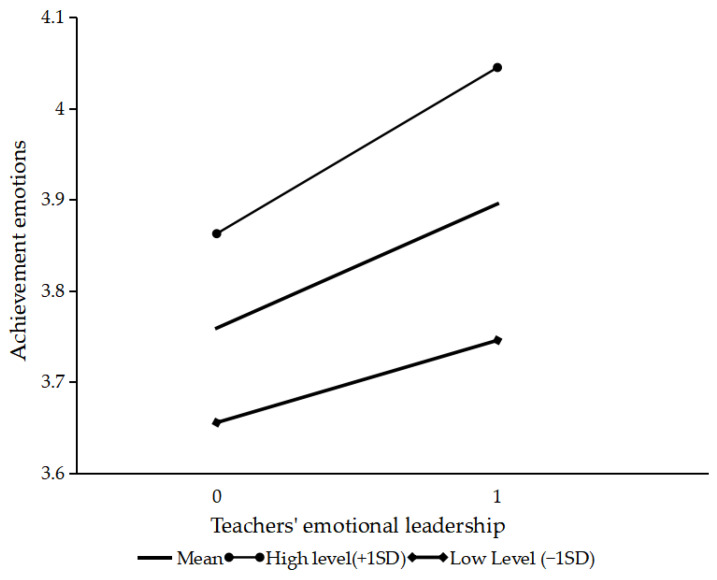
The moderating role of emotional susceptibility.

Table 4 indicates that both teachers’ emotional leadership (*B* = 0.136, *p* < 0.01) and emotional susceptibility (*B* = 0.102, *p* < 0.01) were significant predictors of achievement emotions. Importantly, the interaction term between teachers’ emotional leadership and emotional susceptibility was also significant (*B* = 0.045, *p* < 0.05), suggesting that the relationship between teachers’ emotional leadership and achievement emotions was moderated by emotional susceptibility. Coupled with Figure 3, the slope at high levels of emotional susceptibility was higher than at low levels, indicating that when emotional susceptibility was high, the impact of teachers’ emotional leadership on achievement emotions was stronger. Hypothesis 3 is thus supported.

### 4.6. Moderated Mediation Effect Test

Table 5 demonstrates that the mediating effect of achievement emotions existed across varying levels of emotional susceptibility. Specially, at the low level, the confidence interval ranged from 0.001 to 0.035, excluding zero, which signified a significant mediating effect (effect size = 0.019). At the mean level, the confidence interval spanned from 0.014 to 0.042, also excluding zero, corroborating a significant mediating effect (effect size = 0.029). At the high level, the confidence interval extended from 0.015 to 0.059, confirming a significant mediating effect (effect size = 0.038). These results indicate that achievement emotions acted as a mediator regardless of whether emotional susceptibility was at a low, mean, or high level. Additionally, Table 6 indicates that the index of moderated mediation was 0.009 and the confidence intervals included zero, suggesting that there was no significant moderated mediation effect. Hypothesis 4 was not supported.

## 5. Discussion

The first research question examined the impact of teachers’ emotional leadership on college students’ learning engagement, revealing a significant positive effect. This finding is similar to that of Gunasekara et al. [29], which highlighted the beneficial influence of teachers’ emotional intelligence on college students’ learning engagement. Potential reasons contributing to the enhancement of learning engagement through teachers’ emotional leadership include its ability to foster positive teacher–student relationships and to fulfill the psychological needs of college students. Teachers who demonstrate emotional leadership possess a deeper understanding and empathy towards the experiences and needs of college students [58], enabling them to provide greater support and affective connections in teacher–student interactions [59]. These enhanced emotional bonds contribute to a positive teacher–student relationship, which enhances learning engagement [60]. Additionally, teachers with emotional leadership are particularly effective at addressing the psychological needs of college students, which include autonomy, competence, and relatedness, thus improving learning engagement [61]. By providing emotional support, teachers empower college students to make choices that reflect their personal goals and interests, thereby enhancing their sense of autonomy [62]. Emotional leadership also helps teachers acknowledge college students’ achievements, provide constructive feedback, and offer challenges that match college students’ skill levels, increasing their sense of competence [62]. Teachers with strong emotional leadership foster a supportive learning environment where college students feel valued and understood, which satisfies their need for relatedness [63]. In summary, by nurturing positive teacher–student relationships and meeting psychological needs through emotional leadership, teachers significantly enhance college students’ learning engagement. Given the profound impact of teachers’ emotional leadership on learning engagement, it is imperative for teachers to enhance their emotional self-awareness. Teachers should accurately identify and comprehend their own emotional states and understand how these emotions affect their teaching practices and learning engagement. During the instructional process, teachers should manage their emotional expressions effectively by controlling emotional fluctuations and maintaining a positive, encouraging demeanor. Furthermore, they should convey their emotions through appropriate social skills and demonstrate empathy to establish emotional connections with their students. Teachers should also remain vigilant regarding the emotional well-being of their students, promptly addressing any emotional issues with suitable support and interventions. By adopting these strategies, teachers can enhance college students’ emotional resonance and learning motivation, significantly boosting their engagement in the learning process.

The second research question explores the impact of teachers’ emotional leadership on college students’ learning engagement, mediated by achievement emotions. The findings reveal that teachers’ emotional leadership enhances college students’ achievement emotions, and subsequently improves their engagement in learning activities. This mirrors the findings reported by Sadoughi and Hejazi, indicating that both teacher support and positive achievement emotions are significantly correlated with enhanced learning engagement among college students, with achievement emotions mediating this relationship [39]. Similarly, Liu et al. discovered that teacher support significantly boosts learning engagement in mathematics, as also mediated by achievement emotions [64]. The current finding underscores the important role of teachers’ emotional leadership in shaping college students’ achievement emotions and learning engagement. This influence is crucial as it aligns college students’ achievement emotions with the learning activities, thereby preparing them for more effective learning. Teachers who employ effective strategies for the recognition, identification, and management of achievement emotions not only provide support, but also create the necessary emotional valence for college students. This enables college students to assess their learning activities positively, which in turn promotes deep engagement at cognitive, emotional, and behavioral levels [65,66]. The mediating role of achievement emotions suggests two potential pathways for improving the teaching and learning process. On the one hand, teachers are encouraged to engage in emotional role modeling for college students. They can share their personal experiences with specific emotions encountered during learning, and their coping strategies. This approach could enhance college students’ perceptions of control and value concerning their learning activities, which, in turn, boosts their motivation and participation in learning. On the other hand, it is imperative for teachers to build strong emotional connections with college students, as these connections can significantly impact their achievement emotions. Such connections can be effectively developed by teachers who engage in conversations about everyday matters, actively listen to college students’ experiences with challenges, provide constructive feedback on academic performance, and guide them towards future endeavors. By adopting these strategies, teachers can effectively influence college students’ achievement emotions, which in turn enhances their engagement with and performance in learning activities.

The third research question investigates the moderating role of emotional susceptibility in the relationship between teachers’ emotional leadership and college students’ achievement emotions. Although only a modest percentage of variance was accounted for in our analysis, the results confirm that emotional susceptibility significantly influences how college students respond to teachers’ emotional leadership. This finding aligns with the AET, which posits that individuals may react differently to the same affective event due to individual traits [18]. Moreover, our study highlights the phenomenon of emotional contagion in higher-education settings [67], demonstrating that college students with high emotional susceptibility are more sensitive to the emotional leadership of their teachers, and consequently exhibit more pronounced emotional reactions. Conversely, college students with lower emotional susceptibility show diminished sensitivity to these cues, leading to less intense emotional responses. This indicates that emotional susceptibility acts as a cognitive filter that influences how emotional information is processed [45], thus significantly affecting college students’ different perceptions and evaluations of their teachers’ emotional leadership. These perceptions and evaluations, in turn, are pivotal in shaping college students’ own emotional reactions. Therefore, it is essential to understand the role of emotional susceptibility in how college students perceive and evaluate teachers’ emotional leadership. These insights underline the importance of developing customized emotional leadership strategies and interventions tailored to the diverse emotional needs of college students, thereby positively influencing their achievement emotions. In light of the moderating role of emotional susceptibility, it is critical to support and assist teachers in assessing the differences in college students’ emotional susceptibility. Providing teachers with emotional recognition software and other artificial intelligence (AI) tools, for example, can enhance their understanding of students’ emotional states. These tools can help teachers identify the behaviors, expressions, and physiological reactions of college students with varying levels of emotional susceptibility in large classroom settings, thus positively influencing students’ achievement emotions and learning engagement. However, it is crucial to address both ethical and practical considerations. Obtaining college students’ consent before using these tools is imperative, along with ensuring transparency about how the data will be collected, stored, and used. Moreover, teachers should receive proper training to responsibly interpret AI-generated insights and ethically integrate these insights into their teaching practices.

The last research question investigates whether emotional susceptibility moderates the mediating effect of teachers’ emotional leadership on college students’ learning engagement through achievement emotions. The results indicate that emotional susceptibility influences the relationship between teachers’ emotional leadership and college students’ learning engagement through achievement emotions at varying levels. However, the differences in the mediating effects across levels of emotional susceptibility do not vary significantly, which is inconsistent with our initial hypothesis. The discrepancy between the significant mediation effects at individual levels and the non-significant moderated mediation suggests that the influence of teachers’ emotional leadership on college students’ learning engagement through achievement emotions remains consistent across different levels of emotional susceptibility within the student population. According to AET [18], variations in individual traits related to emotional reactions, such as emotional susceptibility, may either amplify or mitigate the impacts of leadership behaviors within workplace environments. Furthermore, the extent of these effects is not only determined by individual traits, but also by the atmosphere that elicits these emotional reactions [18]. In educational settings, teachers’ emotional leadership helps to create a uniformly positive and supportive atmosphere that similarly impacts all college students. This uniformity could diminish the differential effects of emotional susceptibility, thereby explaining the non-significant results for moderated mediation observed statistically. This implies that the variability in emotional susceptibility might be overshadowed by the atmosphere, leading to a homogenized response to the emotional leadership exhibited by teachers across the college student body. Moreover, according to control–value theory [32], there are individual and atmospheric antecedents that can influence resulting achievement emotions. The achievement emotions among college students are stimulated by teachers’ emotional leadership and intensified by the atmosphere. As achievement emotions play a crucial role in learning engagement, they may standardize the emotional responses of college students with varying emotional susceptibility and mask the potential moderating role of emotional susceptibility. Additionally, the consistent or homogenous emotional responses of college students exemplify group affective tone. Group affective tone refers to the collective emotional climate that emerges from the aggregation of individual members’ emotions within the group [68]. When group members encounter the same affective events, they tend to experience similar emotions [68]. Teachers and college students constitute a learning group. Within this group, teachers’ emotional leadership creates the same affective events, which shapes the collective emotions of college students. In this case, the impact of emotional susceptibility on learning engagement might be supplanted by the effects of group emotions. This finding aligns with the understanding that individual traits and the eliciting atmosphere are critical in shaping emotional reactions and outcomes. Practically, if teachers’ emotional leadership is consistent and potent, it could exert a similar motivational effect on all college students, thus reducing the variance in emotional susceptibility impacts among different college students. Such consistency is likely achieved through the establishment of a positive and supportive atmosphere and active emotional leadership practices, rendering individual differences less significant under the overarching influence of the atmosphere.

The findings of this study underscore the complex interplay among emotional factors and their collective impact on learning engagement, and provide insights into how higher education institutions and teachers can leverage emotional factors to enhance learning engagement effectively. Practically, in the selection and appointment of teachers, emotional leadership capabilities such as empathy, emotional contagion, and appropriate emotional strategies should be considered important criteria for assessment and promotion. Prioritizing those who can invest emotional resources in teaching and support student development is essential when joining the higher education industry. Regarding teacher training, studies have indicated that targeted training programs can improve teachers’ empathic concern and emotional intelligence, thereby positively influencing their teaching practices [69,70]. Therefore, higher education institutions should provide training programs and strengthen support for teachers’ emotional leadership abilities. Teachers can strategically use their knowledge of emotional leadership, as well as college students’ achievement emotions and emotional susceptibility, to design interventions that maximize positive learning outcomes. For instance, teachers can tailor their emotional leadership strategies to create a classroom environment that not only acknowledges but actively engages with the varying levels of emotional susceptibility among college students. By integrating this understanding into their teaching practices, teachers can proactively address and leverage college students’ achievement emotions. This could involve structuring classroom interactions and feedback in a way that reinforces positive emotions like pride and enjoyment, especially after accomplishments, and mitigating negative emotions following setbacks. These activities help college students better manage their emotions, leading to sustained learning engagement and improved learning outcomes.

## 6. Conclusions, Limitations and Future Research

This study explored the dynamics between teachers’ emotional leadership and learning engagement among 1034 Chinese college students. Regression analyses revealed that teachers’ emotional leadership has a significant positive impact on college students’ learning engagement, exerting both direct and indirect effects, with the latter mediated through college students’ achievement emotions. Additionally, college students’ emotional susceptibility was not found to moderate these mediating effects. These findings validate the universal applicability of AET in higher education, highlighting the pivotal role of complex emotional interactions in shaping educational experiences. Practically, these insights suggest that higher education institutions should prioritize emotional leadership in teacher selection, training, and development. Moreover, fostering a supportive learning atmosphere and building strong emotional connections with college students are crucial strategies for teachers. Utilizing tools like emotional recognition software while considering ethical implications can further aid teachers in understanding and responding to college students’ emotional needs. These strategies not only enhance the educational experience, but also significantly increase college students’ involvement in and commitment to their academic pursuits.

This study has certain limitations. First, the sample of this study comes from Chinese universities. According to Mesquita and Frijda, cultural variations in emotions are vital [71]. It suggests that socio-economic backgrounds may affect teachers’ emotional leadership and college students’ learning engagement. Future research could expand the sample range to include teachers and students from different regions, types of universities and disciplines to enhance the representativeness and generalizability of the findings. Additionally, international comparative studies could be considered to explore the relationship between teachers’ emotional leadership and college students’ learning engagement in different cultural contexts.

Second, while our study provides insights into the dynamics of emotional leadership, achievement emotions, emotional susceptibility, and learning engagement, we recognize that these are only a subset of the myriad factors impacting learning engagement. Learning engagement is a complex construct influenced by a diverse array of elements. These include intrinsic factors such as learning motivation and self-efficacy, as well as extrinsic factors including the teacher’s expertise level, college students’ prior educational experiences, class size, course type, the specific content being taught, and the prevailing teaching models. Future research should therefore expand the scope of investigation to encompass these additional variables, allowing for a more comprehensive understanding of what influences learning engagement.

Third, the survey administration process itself posed limitations. The survey was conducted over a short period from January to February 2024, which may have introduced temporal biases due to specific academic pressures during this period. Future research should consider using longitudinal study designs to capture a more representative sample of college students’ experiences across different academic phases, and reduce potential temporal biases.

Lastly, a significant limitation is the absence of behavioral observations of teachers and college students. While self-report surveys are convenient, they do not establish causal relationships. Future studies should incorporate direct behavioral observations to examine potential causal links among the variables discussed. Specifically, methodologies such as classroom observations, where researchers can systematically record teacher–student interactions and emotional exchanges, could be employed.

## Figures and Tables

**Table 1:** Results of normality test.

Variable	N	Skewness	Kurtosis
Learning engagement	1034	−0.087	−0.131
Teachers’ emotional leadership	1034	−0.958	0.369
Achievement emotions	1034	−2.680	5.606
Emotional susceptibility	1034	−0.589	−0.373

**Table 2:** Correlation coefficients.

Variable	M	SD	Learning Engagement	Teachers’ Emotional Leadership	Achievement Emotions	Emotional Susceptibility
Learning engagement	3.822	0.771	1			
Teachers’ emotional leadership	3.894	1.020	0.395 **	1		
Achievement emotions	3.778	0.723	0.277 **	0.229 **	1	
Emotional susceptibility	3.510	1.107	0.371 **	0.383 **	0.213 **	1

** *p* < 0.01.

**Table 3:** Unstandardized coefficients for testing the direct and mediating effects (N = 1034).

	Symbol	Meaning	Effect Size	95% CI	SE	*p*
Teachers’ emotional leadership=>Achievement emotions=>Learning engagement	a × b	Indirect effect	0.034 **	[0.027, 0.066]	0.01	0.000
Teachers’ emotional leadership=>Achievement emotions	a	X=>M	0.162 **	[0.120, 0.204]	0.021	0.000
Achievement emotions=>Learning engagement	b	M=>Y	0.210 **	[0.150, 0.270]	0.031	0.000
Teachers’ emotional leadership=>Learning engagement	c′	Direct effect	0.264 **	[0.222, 0.307]	0.022	0.000
Teachers’ emotional leadership=>Learning engagement	c	Total Effect	0.298 **	[0.256, 0.341]	0.022	0.000

** *p* < 0.01. X: Teachers’ emotional leadership. M: Achievement emotions. Y: Learning engagement. Bootstrap sample = 1000; 95% CI reported as [lower limit confidence interval, upper limit confidence interval].

**Table 4:** Moderating effect test (N = 1034).

	Model				
	*B*	SE	*t*	*p*	*β*
Constant	3.759 **	0.023	163.893	0.000	-
Teachers’ emotional leadership	0.136 **	0.024	5.747	0.000	0.192
Emotional susceptibility	0.102 **	0.023	4.418	0.000	0.143
Teachers’ emotional leadership × Emotional susceptibility	0.045 *	0.019	2.366	0.018	0.073
VIF	1.070				
*R* squared	0.076				
Adjusted *R* squared	0.073				
*F*	*F* (3, 1030) = 28.150, *p* = 0.000				
Δ*R* squared	0.005				
Δ*F*	*F* (1, 1030) = 5.596, *p* = 0.018				

Dependent variable: achievement emotions. * *p* < 0.05. ** *p* < 0.01.

**Table 5:** Conditional indirect effects.

Mediator	Level of Emotional Susceptibility	Level Value	Effect	SE (Boot)	95% LLCI	95% ULCI
Achievement emotions	Low level (−1SD)	2.492	0.019	0.009	0.001	0.035
	Mean	3.509	0.029	0.007	0.014	0.042
	High level (+1SD)	4.527	0.038	0.011	0.015	0.059

LLCI: lower limit confidence interval. ULCI: upper limit confidence interval.

**Table 6:** Index of moderated mediation.

Moderator	Mediator	Index	SE (Boot)	95% LLCI	95% ULCI
Emotional susceptibility	Achievement emotions	0.009	0.007	−0.003	0.023

LLCI: lower limit confidence interval. ULCI: upper limit confidence interval.

## Data Availability

Research data of this study can be obtained from the corresponding author upon reasonable request.

## Appendix A

**Table A1:** Item statistics for the scale measuring teachers’ emotional leadership.

Items	Mean	SE	CITC	Item-Deleted α	α
TEL 1	3.759	1.136	0.892	0.969	0.973
TEL 2	3.792	1.124	0.904	0.968	
TEL 3	4.022	1.098	0.888	0.969	
TEL 4	3.826	1.101	0.913	0.967	
TEL 5	3.983	1.088	0.912	0.968	
TEL 6	3.894	1.079	0.912	0.968	
TEL 7	3.983	1.075	0.881	0.970	

**Table A2:** Inter-item correlation matrix of the scale measuring teachers’ emotional leadership.

Items	TEL 1	TEL 2	TEL 3	TEL 4	TEL 5	TEL 6	TEL 7
TEL 1	1.000	0.878	0.796	0.861	0.810	0.838	0.793
TEL 2	0.878	1.000	0.815	0.869	0.827	0.849	0.798
TEL 3	0.796	0.815	1.000	0.823	0.864	0.822	0.842
TEL 4	0.861	0.869	0.823	1.000	0.850	0.870	0.805
TEL 5	0.810	0.827	0.864	0.850	1.000	0.868	0.862
TEL 6	0.838	0.849	0.822	0.870	0.868	1.000	0.828
TEL 7	0.793	0.798	0.842	0.805	0.862	0.828	1.000

## Appendix B

**Table A3:** Item statistics for the scale measuring achievement emotions.

Items	Mean	SE	CITC	Item-Deleted α	α
AE 1	3.793	1.045	0.639	0.962	0.963
AE 2	3.810	1.059	0.669	0.961	
AE 3	3.786	1.079	0.652	0.961	
AE 4	3.750	1.045	0.641	0.961	
AE 5	3.787	1.053	0.647	0.961	
AE 6	3.798	1.059	0.658	0.961	
AE 7	3.710	1.037	0.659	0.961	
AE 8	3.781	1.063	0.656	0.961	
AE 9	3.776	1.071	0.650	0.961	
AE 10	3.746	1.057	0.646	0.961	
AE 11	3.771	1.071	0.646	0.961	
AE 12	3.786	1.039	0.657	0.961	
AE 13	3.817	1.062	0.648	0.961	
AE 14	3.766	1.058	0.632	0.962	
AE 15	3.774	1.079	0.648	0.961	
AE 16	3.754	1.051	0.651	0.961	
AE 17	3.806	1.072	0.673	0.961	
AE 18	3.764	1.066	0.656	0.961	
AE 19	3.774	1.057	0.650	0.961	
AE 20	3.810	1.072	0.659	0.961	
AE 21	3.770	1.092	0.672	0.961	
AE 22	3.788	1.065	0.658	0.961	
AE 23	3.794	1.050	0.632	0.962	
AE 24	3.726	1.046	0.650	0.961	
AE 25	3.762	1.071	0.668	0.961	
AE 26	3.789	1.059	0.647	0.961	
AE 27	3.785	1.061	0.677	0.961	
AE 28	3.751	1.067	0.652	0.961	
AE 29	3.774	1.068	0.669	0.961	
AE 30	3.777	1.047	0.670	0.961	
AE 31	3.788	1.072	0.658	0.961	
AE 32	3.795	1.097	0.654	0.961	

**Table A4:** Inter-item correlation matrix of the scale measuring achievement emotions.

Items	AE 1	AE 2	AE 3	AE 4	AE 5	AE 6	AE 7	AE 8	AE 9	AE 10	AE 11	AE 12	AE 13	AE 14	AE 15	AE 16	AE 17	AE 18	AE 19	AE 20	AE 21	AE 22	AE 23	AE 24	AE 25	AE 26	AE 27	AE 28	AE 29	AE 30	AE 31	AE 32
AE 1	1.000	0.414	0.425	0.457	0.425	0.417	0.481	0.472	0.426	0.436	0.432	0.409	0.430	0.411	0.462	0.401	0.434	0.421	0.413	0.420	0.439	0.428	0.457	0.455	0.458	0.434	0.484	0.442	0.419	0.456	0.409	0.424
AE 2	0.414	1.000	0.440	0.413	0.433	0.454	0.425	0.487	0.475	0.447	0.455	0.449	0.456	0.453	0.466	0.441	0.497	0.457	0.463	0.448	0.487	0.452	0.404	0.467	0.458	0.471	0.451	0.460	0.494	0.487	0.441	0.471
AE 3	0.425	0.440	1.000	0.424	0.451	0.446	0.413	0.406	0.465	0.461	0.449	0.495	0.426	0.424	0.455	0.419	0.475	0.476	0.427	0.449	0.460	0.439	0.432	0.460	0.479	0.437	0.439	0.428	0.450	0.452	0.444	0.415
AE 4	0.457	0.413	0.424	1.000	0.430	0.441	0.461	0.415	0.447	0.419	0.449	0.440	0.405	0.443	0.431	0.419	0.463	0.440	0.449	0.455	0.443	0.432	0.420	0.435	0.438	0.426	0.453	0.420	0.428	0.459	0.463	0.430
AE 5	0.425	0.433	0.451	0.430	1.000	0.441	0.451	0.452	0.407	0.438	0.439	0.440	0.442	0.419	0.431	0.457	0.485	0.444	0.423	0.426	0.468	0.424	0.463	0.463	0.443	0.421	0.428	0.432	0.461	0.436	0.450	0.438
AE 6	0.417	0.454	0.446	0.441	0.441	1.000	0.440	0.454	0.445	0.426	0.415	0.454	0.447	0.424	0.436	0.454	0.454	0.467	0.436	0.472	0.445	0.470	0.439	0.448	0.487	0.494	0.446	0.425	0.465	0.447	0.451	0.443
AE 7	0.481	0.425	0.413	0.461	0.451	0.440	1.000	0.421	0.440	0.442	0.426	0.433	0.450	0.401	0.443	0.462	0.469	0.446	0.465	0.422	0.467	0.458	0.430	0.426	0.479	0.467	0.448	0.455	0.461	0.457	0.467	0.491
AE 8	0.472	0.487	0.406	0.415	0.452	0.454	0.421	1.000	0.448	0.465	0.414	0.441	0.444	0.421	0.420	0.440	0.467	0.432	0.460	0.446	0.436	0.455	0.435	0.454	0.453	0.486	0.461	0.470	0.459	0.441	0.462	0.436
AE 9	0.426	0.475	0.465	0.447	0.407	0.445	0.440	0.448	1.000	0.444	0.481	0.426	0.461	0.437	0.417	0.430	0.442	0.453	0.453	0.458	0.452	0.447	0.411	0.407	0.424	0.414	0.443	0.441	0.473	0.425	0.462	0.455
AE 10	0.436	0.447	0.461	0.419	0.438	0.426	0.442	0.465	0.444	1.000	0.456	0.452	0.444	0.426	0.437	0.446	0.411	0.441	0.448	0.429	0.421	0.439	0.402	0.418	0.464	0.423	0.446	0.450	0.447	0.477	0.439	0.447
AE 11	0.432	0.455	0.449	0.449	0.439	0.415	0.426	0.414	0.481	0.456	1.000	0.426	0.448	0.423	0.443	0.398	0.438	0.455	0.444	0.449	0.466	0.449	0.434	0.451	0.449	0.440	0.465	0.425	0.442	0.437	0.439	0.409
AE 12	0.409	0.449	0.495	0.440	0.440	0.454	0.433	0.441	0.426	0.452	0.426	1.000	0.451	0.446	0.449	0.456	0.478	0.460	0.444	0.483	0.455	0.468	0.429	0.417	0.433	0.434	0.469	0.413	0.453	0.465	0.443	0.459
AE 13	0.430	0.456	0.426	0.405	0.442	0.447	0.450	0.444	0.461	0.444	0.448	0.451	1.000	0.472	0.411	0.420	0.433	0.446	0.441	0.469	0.437	0.435	0.432	0.400	0.455	0.436	0.459	0.445	0.470	0.455	0.430	0.434
AE 14	0.411	0.453	0.424	0.443	0.419	0.424	0.401	0.421	0.437	0.426	0.423	0.446	0.472	1.000	0.436	0.427	0.444	0.446	0.437	0.448	0.435	0.428	0.419	0.459	0.409	0.393	0.436	0.420	0.444	0.442	0.434	0.401
AE 15	0.462	0.466	0.455	0.431	0.431	0.436	0.443	0.420	0.417	0.437	0.443	0.449	0.411	0.436	1.000	0.472	0.466	0.431	0.446	0.449	0.422	0.438	0.427	0.429	0.430	0.452	0.453	0.396	0.464	0.469	0.440	0.452
AE 16	0.401	0.441	0.419	0.419	0.457	0.454	0.462	0.440	0.430	0.446	0.398	0.456	0.420	0.427	0.472	1.000	0.459	0.428	0.433	0.442	0.427	0.458	0.434	0.474	0.482	0.453	0.483	0.436	0.453	0.466	0.430	0.443
AE 17	0.434	0.497	0.475	0.463	0.485	0.454	0.469	0.467	0.442	0.411	0.438	0.478	0.433	0.444	0.466	0.459	1.000	0.450	0.466	0.452	0.472	0.446	0.412	0.460	0.458	0.431	0.476	0.452	0.493	0.490	0.475	0.451
AE 18	0.421	0.457	0.476	0.440	0.444	0.467	0.446	0.432	0.453	0.441	0.455	0.460	0.446	0.446	0.431	0.428	0.450	1.000	0.435	0.455	0.466	0.479	0.421	0.439	0.448	0.436	0.478	0.431	0.455	0.439	0.435	0.444
AE 19	0.413	0.463	0.427	0.449	0.423	0.436	0.465	0.460	0.453	0.448	0.444	0.444	0.441	0.437	0.446	0.433	0.466	0.435	1.000	0.439	0.467	0.452	0.434	0.409	0.448	0.413	0.484	0.441	0.446	0.445	0.424	0.432
AE 20	0.420	0.448	0.449	0.455	0.426	0.472	0.422	0.446	0.458	0.429	0.449	0.483	0.469	0.448	0.449	0.442	0.452	0.455	0.439	1.000	0.458	0.449	0.417	0.408	0.471	0.419	0.446	0.450	0.487	0.471	0.445	0.462
AE 21	0.439	0.487	0.460	0.443	0.468	0.445	0.467	0.436	0.452	0.421	0.466	0.455	0.437	0.435	0.422	0.427	0.472	0.466	0.467	0.458	1.000	0.455	0.448	0.457	0.478	0.462	0.498	0.490	0.447	0.485	0.468	0.458
AE 22	0.428	0.452	0.439	0.432	0.424	0.470	0.458	0.455	0.447	0.439	0.449	0.468	0.435	0.428	0.438	0.458	0.446	0.479	0.452	0.449	0.455	1.000	0.433	0.430	0.454	0.461	0.460	0.438	0.438	0.471	0.454	0.452
AE 23	0.457	0.404	0.432	0.420	0.463	0.439	0.430	0.435	0.411	0.402	0.434	0.429	0.432	0.419	0.427	0.434	0.412	0.421	0.434	0.417	0.448	0.433	1.000	0.472	0.411	0.421	0.450	0.436	0.455	0.425	0.438	0.419
AE 24	0.455	0.467	0.460	0.435	0.463	0.448	0.426	0.454	0.407	0.418	0.451	0.417	0.400	0.459	0.429	0.474	0.460	0.439	0.409	0.408	0.457	0.430	0.472	1.000	0.442	0.422	0.450	0.448	0.429	0.452	0.465	0.481
AE 25	0.458	0.458	0.479	0.438	0.443	0.487	0.479	0.453	0.424	0.464	0.449	0.433	0.455	0.409	0.430	0.482	0.458	0.448	0.448	0.471	0.478	0.454	0.411	0.442	1.000	0.453	0.464	0.438	0.460	0.465	0.475	0.477
AE 26	0.434	0.471	0.437	0.426	0.421	0.494	0.467	0.486	0.414	0.423	0.440	0.434	0.436	0.393	0.452	0.453	0.431	0.436	0.413	0.419	0.462	0.461	0.421	0.422	0.453	1.000	0.481	0.457	0.453	0.411	0.436	0.435
AE 27	0.484	0.451	0.439	0.453	0.428	0.446	0.448	0.461	0.443	0.446	0.465	0.469	0.459	0.436	0.453	0.483	0.476	0.478	0.484	0.446	0.498	0.460	0.450	0.450	0.464	0.481	1.000	0.497	0.467	0.466	0.456	0.441
AE 28	0.442	0.460	0.428	0.420	0.432	0.425	0.455	0.470	0.441	0.450	0.425	0.413	0.445	0.420	0.396	0.436	0.452	0.431	0.441	0.450	0.490	0.438	0.436	0.448	0.438	0.457	0.497	1.000	0.435	0.467	0.449	0.475
AE 29	0.419	0.494	0.450	0.428	0.461	0.465	0.461	0.459	0.473	0.447	0.442	0.453	0.470	0.444	0.464	0.453	0.493	0.455	0.446	0.487	0.447	0.438	0.455	0.429	0.460	0.453	0.467	0.435	1.000	0.469	0.436	0.446
AE 30	0.456	0.487	0.452	0.459	0.436	0.447	0.457	0.441	0.425	0.477	0.437	0.465	0.455	0.442	0.469	0.466	0.490	0.439	0.445	0.471	0.485	0.471	0.425	0.452	0.465	0.411	0.466	0.467	0.469	1.000	0.473	0.432
AE 31	0.409	0.441	0.444	0.463	0.450	0.451	0.467	0.462	0.462	0.439	0.439	0.443	0.430	0.434	0.440	0.430	0.475	0.435	0.424	0.445	0.468	0.454	0.438	0.465	0.475	0.436	0.456	0.449	0.436	0.473	1.000	0.451
AE 32	0.424	0.471	0.415	0.430	0.438	0.443	0.491	0.436	0.455	0.447	0.409	0.459	0.434	0.401	0.452	0.443	0.451	0.444	0.432	0.462	0.458	0.452	0.419	0.481	0.477	0.435	0.441	0.475	0.446	0.432	0.451	1.000

## Appendix C

**Table A5:** Item statistics for the scale measuring emotional susceptibility.

Item	Mean	SE	CITC	Item-Deleted α	α
ES 1	3.323	1.213	0.743	0.969	0.970
ES 2	3.572	1.180	0.822	0.967	
ES 3	3.709	1.186	0.873	0.966	
ES 4	3.654	1.192	0.865	0.966	
ES 5	3.559	1.255	0.835	0.967	
ES 6	3.337	1.165	0.786	0.968	
ES 7	3.355	1.184	0.812	0.968	
ES 8	3.498	1.220	0.840	0.967	
ES 9	3.365	1.127	0.827	0.967	
ES 10	3.749	1.168	0.864	0.966	
ES 11	3.414	1.227	0.813	0.968	
ES 12	3.430	1.143	0.843	0.967	
ES 13	3.658	1.182	0.863	0.966	

**Table A6:** Inter-item correlation matrix of the scale measuring emotional susceptibility.

Item	ES 1	ES 2	ES 3	ES 4	ES 5	ES 6	ES 7	ES 8	ES 9	ES 10	ES 11	ES 12	ES 13
ES 1	1.000	0.670	0.684	0.700	0.622	0.607	0.663	0.596	0.646	0.614	0.585	0.661	0.659
ES 2	0.670	1.000	0.836	0.783	0.693	0.631	0.650	0.676	0.663	0.801	0.663	0.673	0.726
ES 3	0.684	0.836	1.000	0.840	0.747	0.663	0.679	0.719	0.695	0.859	0.705	0.719	0.805
ES 4	0.700	0.783	0.840	1.000	0.738	0.680	0.705	0.721	0.696	0.807	0.683	0.736	0.784
ES 5	0.622	0.693	0.747	0.738	1.000	0.635	0.643	0.851	0.679	0.752	0.815	0.687	0.717
ES 6	0.607	0.631	0.663	0.680	0.635	1.000	0.793	0.665	0.762	0.653	0.631	0.731	0.684
ES 7	0.663	0.650	0.679	0.705	0.643	0.793	1.000	0.673	0.797	0.663	0.629	0.767	0.721
ES 8	0.596	0.676	0.719	0.721	0.851	0.665	0.673	1.000	0.692	0.752	0.861	0.696	0.721
ES 9	0.646	0.663	0.695	0.696	0.679	0.762	0.797	0.692	1.000	0.695	0.664	0.794	0.735
ES 10	0.614	0.801	0.859	0.807	0.752	0.653	0.663	0.752	0.695	1.000	0.719	0.742	0.809
ES 11	0.585	0.663	0.705	0.683	0.815	0.631	0.629	0.861	0.664	0.719	1.000	0.690	0.720
ES 12	0.661	0.673	0.719	0.736	0.687	0.731	0.767	0.696	0.794	0.742	0.690	1.000	0.779
ES 13	0.659	0.726	0.805	0.784	0.717	0.684	0.721	0.721	0.735	0.809	0.720	0.779	1.000

## Appendix D

**Table A7:** Item statistics for the scale measuring learning engagement.

Item	Mean	SE	CITC	Item-Deleted α	α
LE 1	3.755	0.894	0.840	0.976	0.977
LE 2	3.694	0.897	0.849	0.976	
LE 3	3.844	0.854	0.861	0.976	
LE 4	3.877	0.851	0.881	0.975	
LE 5	3.693	0.940	0.818	0.976	
LE 6	3.790	0.901	0.869	0.975	
LE 7	3.779	0.886	0.866	0.976	
LE 8	3.913	0.842	0.868	0.975	
LE 9	3.833	0.873	0.889	0.975	
LE 10	3.848	0.866	0.891	0.975	
LE 11	3.880	0.866	0.864	0.976	
LE 12	3.815	0.894	0.850	0.976	
LE 13	3.874	0.859	0.858	0.976	
LE 14	3.905	0.849	0.823	0.976	

**Table A8:** Inter-item correlation matrix of the scale measuring learning engagement.

Item	LE 1	LE 2	LE 3	LE 4	LE 5	LE 6	LE 7	LE 8	LE 9	LE 10	LE 11	LE 12	LE 13	LE 14
LE 1	1.000	0.892	0.811	0.800	0.733	0.735	0.763	0.717	0.761	0.728	0.689	0.664	0.697	0.628
LE 2	0.892	1.000	0.806	0.805	0.737	0.766	0.760	0.727	0.770	0.755	0.682	0.687	0.688	0.644
LE 3	0.811	0.806	1.000	0.838	0.741	0.756	0.754	0.740	0.772	0.762	0.732	0.699	0.739	0.696
LE 4	0.800	0.805	0.838	1.000	0.738	0.810	0.749	0.787	0.784	0.806	0.750	0.730	0.752	0.716
LE 5	0.733	0.737	0.741	0.738	1.000	0.805	0.741	0.687	0.734	0.736	0.703	0.704	0.672	0.652
LE 6	0.735	0.766	0.756	0.810	0.805	1.000	0.774	0.788	0.785	0.815	0.739	0.729	0.725	0.701
LE 7	0.763	0.760	0.754	0.749	0.741	0.774	1.000	0.783	0.873	0.805	0.758	0.726	0.733	0.684
LE 8	0.717	0.727	0.740	0.787	0.687	0.788	0.783	1.000	0.808	0.843	0.770	0.763	0.751	0.763
LE 9	0.761	0.770	0.772	0.784	0.734	0.785	0.873	0.808	1.000	0.852	0.781	0.746	0.762	0.725
LE 10	0.728	0.755	0.762	0.806	0.736	0.815	0.805	0.843	0.852	1.000	0.773	0.782	0.759	0.750
LE 11	0.689	0.682	0.732	0.750	0.703	0.739	0.758	0.770	0.781	0.773	1.000	0.858	0.848	0.803
LE 12	0.664	0.687	0.699	0.730	0.704	0.729	0.726	0.763	0.746	0.782	0.858	1.000	0.828	0.825
LE 13	0.697	0.688	0.739	0.752	0.672	0.725	0.733	0.751	0.762	0.759	0.848	0.828	1.000	0.871
LE 14	0.628	0.644	0.696	0.716	0.652	0.701	0.684	0.763	0.725	0.750	0.803	0.825	0.871	1.000
